# Neuronal Code for Episodic Time in the Lateral Entorhinal Cortex

**DOI:** 10.3389/fnint.2022.899412

**Published:** 2022-04-29

**Authors:** Kaori Takehara-Nishiuchi

**Affiliations:** ^1^Department of Psychology, University of Toronto, Toronto, ON, Canada; ^2^Department of Cell and Systems Biology, University of Toronto, Toronto, ON, Canada; ^3^Neuroscience Program, University of Toronto, Toronto, ON, Canada

**Keywords:** episodic memory, hippocampus, neocortex, neural activity, electrophysiology, rodents

## Introduction

When we think of how several events unfold during an experience, our sense of the duration, intervals, and order of these events is subjective and distinct from a precise representation of metric time. Such temporal information, so-called episodic time, is fundamental for encoding and retrieving episodic memories, the record of what happened, where it happened, and when. Recently, several studies monitored the activity of individual neurons (Tsao et al., 2018; Bright et al., 2020) or hemodynamics (Bellmund et al., 2019; Montchal et al., 2019) to provide evidence that the neural representation of episodic time exists in the lateral entorhinal cortex (LEC).

The LEC is one of the first and most severely affected regions in Alzheimer's disease patients (Khan et al., 2014; Wisse et al., 2014; Nosheny et al., 2019), and the targeted disruption of the LEC impairs various rodent models of episodic memory (Morrissey and Takehara-Nishiuchi, 2014). Within the network of inter-connected regions supporting episodic memory, the LEC is unique in that it maintains reciprocal connections with the hippocampus on the one hand and with the neocortical regions on the other (Bota et al., 2015; Nilssen et al., 2019). This anatomical feature led to a view that the LEC is an essential relay structure between the hippocampus and neocortex (Squire, 1992; Eichenbaum, 2000, 2017). Specifically, during memory encoding, the LEC routes multiple pieces of highly processed sensory information from the neocortex to the hippocampus, thereby helping the hippocampus combine them into a single representation of an experience. During memory retrieval, the LEC transfers the stored information from the hippocampus to the neocortex to reinstate the neocortical activity patterns during the original experience.

**Figure 1:**
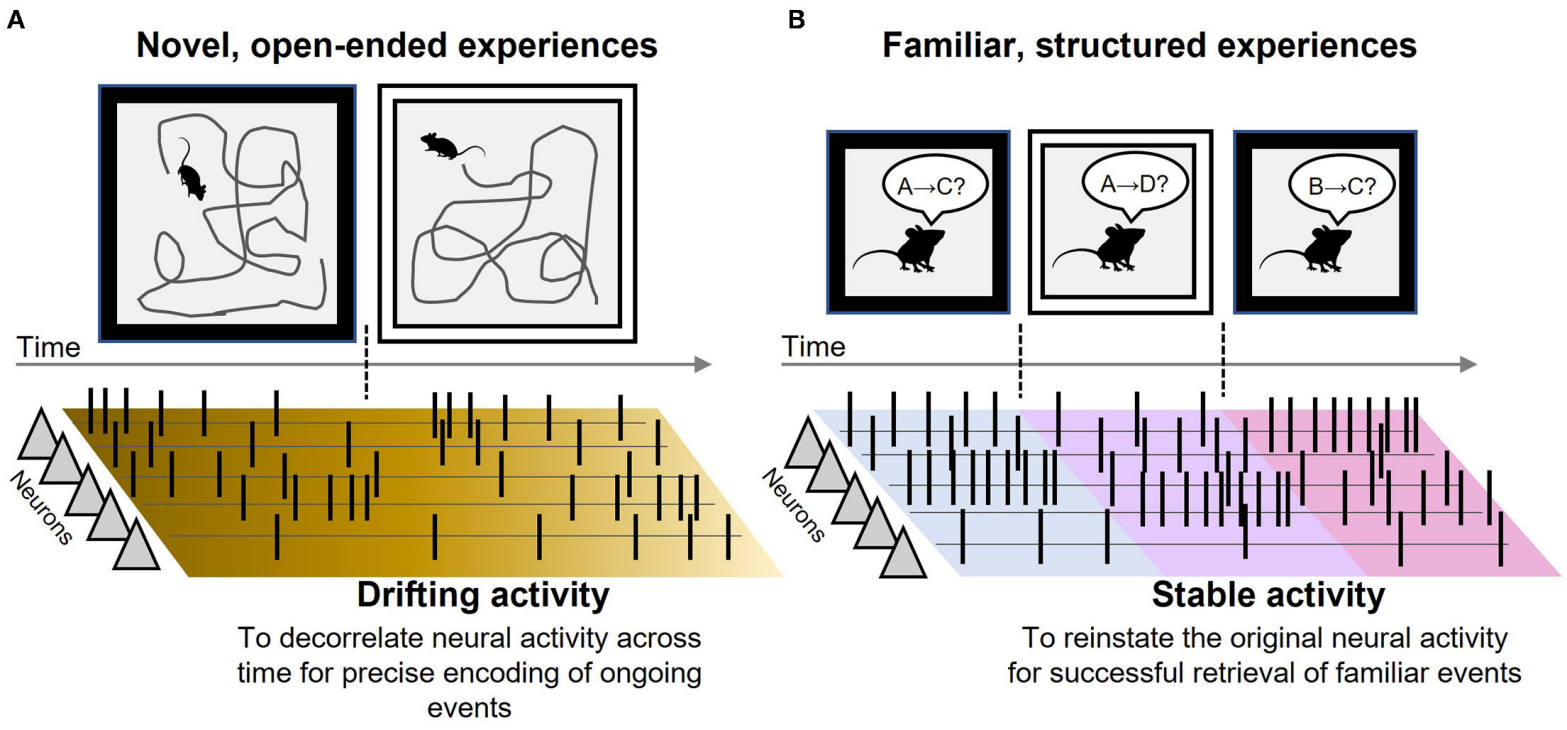
Drifting and stable neural activity in the LEC. **(A)** Schematic depictions of the main findings in Tsao et al. (2018). When a rat sequentially explored two rooms with two different wall colors, some LEC neurons gradually increased/decreased the rate of action potentials (black vertical lines). Because the time constant of the changing rates differed across neurons, the population activity pattern continuously changed across time and did not repeat the identical pattern. Such ever-drifting activity helps efferent regions decorrelate the neural activity across time, thereby putting a unique timestamp on each ongoing event during a novel experience. **(B)** Schematic depictions of the main findings in Pilkiw et al. (2017). Over two weeks, a rat repeatedly underwent multiple combinations of stimulus pairings (A–D) in two different environments in a fixed temporal order. Almost all LEC neurons maintained stable rates of action potentials while the rat was experiencing one of the combinations; however, they drastically changed the rates upon the transition from one combination to another. Such remapping of baseline firing rates generates a unique population activity pattern for each event-environment combination. By stably maintaining the unique pattern during a familiar experience, the LEC helps efferent regions faithfully reinstate the original neural activity pattern associated with familiar events.

The discovery of neural representations of episodic time in the LEC provides a prime opportunity to refine the role of the LEC in the network processes supporting episodic memory. The present opinion article first summarizes findings in several electrophysiological studies that monitored the activity of individual neurons in the LEC with the focus on temporal features of their firing patterns. The survey has identified two distinct types, (1) drifting patterns during novel, open-ended experiences and (2) stable patterns during familiar, structured experiences (Figure 1). I then discuss their potential functions in the network dynamics supporting memory encoding and retrieval with several ideas for future investigations.

## LEC Ensemble Dynamics During Novel, Open-Ended Experiences

Tsao et al. (2018) is the first to demonstrate that LEC neuronal ensembles play an essential role in representing episodic time. They recorded the spiking activity of individual neurons in the LEC while rats were placed in one of two empty boxes with different wall colors (Figure 1A). The rats alternatively visited these boxes 12 times, during which they freely explored in the box for ~four minutes. They found that ~20% of individual LEC neurons monotonically changed their firing rates over time, thereby showing “ramp-like” firing patterns. Importantly, these “temporal cells” marginally changed their firing rates depending on which box the rat was in (selectivity for wall color) or where in the box the rat was at a given time (selectivity for position). In some temporal cells, the ramp started from the box entry and ended with the box exit. In contrast, others showed ramp-like firing patterns covering from the start to the end of the entire recording session. Due to this variation in the time constant of the ramp, LEC ensemble activity patterns differentiated successive visits to the same box, thereby forming an accurate code for the temporal context of the entire experience.

Similar time selectivity was also observed in the entorhinal cortex while head-fixed monkeys passively viewed various images on a computer screen (Bright et al., 2020). Approximately 30% of entorhinal cells changed their firing rates at the onset of the image presentation and returned to their baseline firing rates over variable time durations ranging from a few 100 ms to 10 s.

Collectively, these findings suggest that LEC neurons possess the innate propensity to change firing rates in a ramp-like manner starting from the onset of events, such as an entry to an enclosure or a presentation of novel stimuli. Because the slope of the ramp varies across neurons, neural population activity drastically decorrelates over time, which enables the LEC to represent the passage of time within each experience.

## LEC Ensemble Dynamics During Familiar, Structured Experiences

Parallel evidence suggests that the dynamics of LEC activity become considerably stabilized during a structured experience that consists of repeated presentations of familiar stimuli or repetitive, stereotypic movements in a familiar environment. For example, when rats ran on a figure-eight maze, LEC neuronal ensemble activity no longer drifted across running laps. Instead, it alternated two distinct patterns, each corresponding to a specific turning direction (Tsao et al., 2018). In parallel, during various forms of associative learning tasks, LEC neurons showed stable firing responses to sensory stimuli (Igarashi et al., 2014; Pilkiw et al., 2017; Suter et al., 2019; Woods et al., 2020; Lee et al., 2021) and objects (Keene et al., 2016) that were repeatedly presented over ~30–60 min. Notably, the across-time stability was detected not only in stimulus-evoked firing rates but also spontaneous firing rates during intervals between stimulus presentations (Pilkiw et al., 2017; Figure 1B). In this study, rats repeatedly underwent six blocks of trials in a fixed temporal order over two weeks. Each of six trial blocks included the repeated presentations of one of two sensory stimuli with or without an aversive outcome in one of two rooms. During the entire duration of each trial block, individual LEC neurons showed highly stable baseline firing rates. Upon the transition from one block to the other block, however, almost all neurons changed baseline firing rates drastically, leading to ensemble firing patterns that clearly differentiated all six trial blocks. Moreover, the ensemble firing pattern for each block emerged immediately after the rat entered the room but before the first stimulus presentation, alluding that the LEC network is capable of prospectively signaling imminent sensory events based on the temporal and environmental context.

Collectively, these findings suggest that during familiar, structured experiences, neural activity in the LEC is highly stable across time and even possesses the ability to prospectively signal upcoming events from the temporal contextual information.

## Functional Implications of Drifting and Stable Firing Patterns in the LEC

The evidence summarized above suggests that the temporal stability of neural activity in the LEC changes dramatically depending on what type of experiences subjects are undergoing (Figure 1). During novel, open-ended experiences, the firing rates of individual neurons change over seconds to hours without any strong relations to moment-to-moment changes in subjects' movement or perceivable features of the environment. Such time-dependent drift is markedly suppressed during familiar, structured experiences. The LEC neurons show consistent firing patterns in response to repeatedly presented stimuli and stable baseline firing rates during memory tasks. The co-existence of drifting and stable neural activity might be because the consistency of sensory inputs across time differs between open-ended and structured experiences (Tsao et al., 2018; Sugar and Moser, 2019). In the former, external incoming inputs change continuously and rarely repeat the same patterns. In contrast, in the latter, the subjects repeatedly undergo a near-identical sequence of sensory inputs.

I here argue an alternative view that the drifting and stable activity in the LEC reflects its role in controlling the balance between flexibility and stability of network dynamics. Accumulating evidence suggests that each brain region is endowed with a mix of stable and changing neural activity patterns across various time scales and magnitude (Clopath et al., 2017; Rule et al., 2019; Mau et al., 2020). The widespread anatomical connectivity (Bota et al., 2015; Nilssen et al., 2019) places the LEC in a strategic position to coordinate the switch between the stable and changing neural activity across brain regions depending on the type of experiences. Specifically, during novel experiences, the slowly drifting neural activity in the LEC decorrelates the activity of neurons in the hippocampus, thereby providing timestamps for accurately encoding the temporal sequence of events as they unfold. In contrast, during familiar experiences, the stable neural activity in the LEC will continuously provide excitatory inputs into a specific set of neurons in the hippocampus and neocortex, thereby facilitating the reinstatement of the neural activity patterns associated with a specific experience. Therefore, representations of episodic time in the LEC are one manifestation of the more fundamental role of the LEC in setting the dynamics for memory encoding and retrieval in the hippocampus-neocortical network.

## Discussion

To further explore the functional relevance of time-sensitive activity in the LEC, the two following points warrant future investigations. Firstly, it is crucial to investigate how drifting neural activity in the LEC contributes to developing neural representations of new experiences in efferent regions. By using the computational modeling approach, Rolls and Mills (2019) argued that the activity of temporal cells in the LEC serves as essential inputs for hippocampal neurons to gain selectivity for a specific short time segment within an experience. These neurons, so-called time cells, form sequential firing patterns during intervals between task events (Pastalkova et al., 2008; MacDonald et al., 2011; Modi et al., 2014), representing their temporal information (Howard et al., 2014; Eichenbaum, 2017). However, it is important to note that time cells were detected while well-trained subjects underwent structured experiences. Therefore, it is necessary to manipulate LEC inputs into the hippocampus and examine how it affects the development of the sequential activity of hippocampal neurons during novel experiences.

Secondly, the relevance of stable activity in the LEC for memory retrieval requires further investigation. In support of the model described above, our recent study showed that reversible, pharmacological inactivation of the LEC impaired the reinstatement of task-related neural firings in one of its primary efferent targets, the medial prefrontal cortex (mPFC; Pilkiw et al., 2022). When male rats underwent multiple epochs of identical stimulus sequences in the same environment, the mPFC maintained a stable ensemble firing pattern across repetitions. With LEC inhibition, the mPFC still formed an ensemble pattern that accurately captured the stimulus sequence within each epoch. However, LEC inhibition markedly disrupted its consistency across the epochs by decreasing the proportion of mPFC neurons that stably maintained firing selectivity for stimulus associations. Although these findings support the idea that the LEC is necessary for the reinstatement of cortical memory representations, the pharmacological inactivation lacks the layer, cell-type, and projection specificity. Therefore, future studies must use a manipulation that disrupts the projections from the LEC to a neocortical region or the hippocampus and investigate the impact on their task-related activity during memory retrieval.

To conclude, the discovery of time-sensitive activity in the LEC represents an essential breakthrough for studying how the brain network processes temporal information during the encoding and retrieval of episodic memory. It also illuminates the need to develop a model that explains mechanisms of these processes by incorporating dynamics of neuronal ensemble activity. Future investigations on this topic are also clinically relevant because they will help explain why the disruption of the LEC with aging and Alzheimer's disease results in severe memory deficits.

## Author Contributions

The author confirms being the sole contributor of this work and has approved it for publication.

## Funding

This work was supported by NSERC Discovery Grant (RGPIN-2020-04479) and CFI Leaders Opportunity Fund (25026; KT-N).

## Conflict of Interest

The author declares that the research was conducted in the absence of any commercial or financial relationships that could be construed as a potential conflict of interest.

## Publisher's Note

All claims expressed in this article are solely those of the authors and do not necessarily represent those of their affiliated organizations, or those of the publisher, the editors and the reviewers. Any product that may be evaluated in this article, or claim that may be made by its manufacturer, is not guaranteed or endorsed by the publisher.
